# The Effect of Exposure to “Exemption” Video Advertisements for Functional Foods: A Randomized Control Study in Japan

**DOI:** 10.3390/healthcare10020345

**Published:** 2022-02-11

**Authors:** Reina Iye, Tsuyoshi Okuhara, Hiroko Okada, Eiko Goto, Emi Furukawa, Takahiro Kiuchi

**Affiliations:** 1 Department of Health Communication, Graduate School of Medicine, The University of Tokyo, 7-3-1 Hongo, Bunkyo-ku, Tokyo 113-8655, Japan; efurukawa-tho@umin.ac.jp; 2 Department of Health Communication, School of Public Health, The University of Tokyo, 7-3-1 Hongo, Bunkyo-ku, Tokyo 113-8655, Japan; okuhara-ctr@umin.ac.jp (T.O.); okadahiroko-tky@umin.ac.jp (H.O.); gotoue-tky@umin.ac.jp (E.G.); tak-kiuchi@umin.ac.jp (T.K.)

**Keywords:** advertisements, functional foods, dietary supplements, compensatory health beliefs, health communication

## Abstract

Previous content analysis of video advertisements for functional foods identified “Exemption” advertisements. “Exemption” advertisements may imply to the audiences that “By taking functional foods, I can adopt unhealthy behaviors or I don’t have to adopt healthy behaviors”. In the context of Compensatory Health Beliefs (CHBs), this study refers to these beliefs as functional foods related to CHBs (FF-CHBs). This study aimed to assess the effects of exposure to “Exemption” advertisements for fat-reduction functional foods on audiences. The main hypothesis is exposure to “Exemption” video advertisements increases participants’ FF-CHBs. Participants (*n*  =  788) were randomly assigned to an intervention group that viewed three video advertisements or a control group and answered online self-administered questionnaires. Intervention videos were three videos with the highest number of views per month from among the “Exemption” video advertisements. Control videos were about how to brew green tea. FF-CHBs was assessed before and after the intervention. The intervention group showed significantly greater FF-CHBs after intervention (mean = 2.37 vs. 2.11, *p* < 0.001, η^2^ = 0.026) compared with the control group. “Exemption” functional foods video advertisements increased FF-CHBs that can lead to adopting unhealthy behaviors and avoiding healthy behaviors. The content of these functional foods video advertisements should be improved to promote public health.

## 1. Introduction

### 1.1. What Are “Exemption” Video Advertisements?

Functional foods are defined as products that contain functional ingredients and claim some health-related effect in this study (e.g., foods for specified health uses (FOSHU) and foods with function claims (FFC) in Japan) [1,2]. Functional foods come in various forms, including pills, beverages, snacks, and fresh foods, and are readily available at convenience stores and supermarkets. The market for functional foods is expected to expand [3,4,5].

However, several public health papers have raised questions about the effectiveness of functional foods [6,7,8]. Nonetheless, functional foods are widely advertised. Some countries put measures on functional foods advertisements (ads) [9,10,11]. The US Federal Trade Commission has warned about weight loss ads and stated that there is no “magic” way to lose weight without a sensible diet and regular exercise [12].

In Japan, consumers are mainly interested in functional food products that claim fat-reduction effects, such as neutral fat, visceral fat, and body fat; the market for products claiming effects on neutral fat and body fat is expected to grow [4,13,14]. Despite the increased placement of video ads due to the expansion of the fifth-generation mobile communication system (5G) [15], few studies have discussed the effects of functional foods video ads, and there has been no inductive analysis of video ads. Therefore, we previously conducted content analysis of Japanese video ads for functional foods that claimed fat-reduction effects [16]. We found that video ads labeled “Exemption” were the most common, and accounted for about one-quarter of the 82 videos analyzed [16]. We defined “Exemption” ads as “Taking the product frees the audience from refraining from binge eating” in the process of our content analysis [16]. In one of these “Exemption” ads, fat-rich dishes were shown, and the main character expressed her resistance to eating them, the main character took the functional food and enjoyed the fat-rich dish. Although not directly described, “Exemption” ads may imply to the audience that “By taking functional foods, I can adopt unhealthy behaviors or I don’t have to adopt healthy behaviors” using various symbolic elements such as images, sounds, facial expressions, voice tones, and the mood of the video. However, even if functional foods are consumed, appropriate lifestyle habits such as a well-balanced diet and moderate physical activity are important. The belief that “By taking functional foods, I can adopt unhealthy behaviors or I don’t have to adopt healthy behaviors” is false from a public health perspective [12,17]. Accordingly, if the belief that “By taking functional foods, I can adopt unhealthy behaviors or I don’t have to adopt healthy behaviors” is activated by exposure to “Exemption” ads, these businesses are creating a false perception in their audiences. Adopting moderate exercise and healthy eating brings many benefits [18,19], therefore, this belief may deprive us of health opportunities.

### 1.2. Theoretical Background

Previous studies explored behavior before or after consumption of functional foods [20,21,22,23]. For example, one study found that participants in the weight-loss supplements condition consumed more nougat and preferred more sugar in tapioca than those in the control condition [21]. That study suggested this may be because weight-loss supplements increased the perception of approaching a weight-loss goal and gave permission for self-indulgence; that is, they worked as a “get-out-of-jail-free” card for self-indulgence [21]. In other words, these studies imply the belief that “By taking functional foods, I can adopt unhealthy behaviors or I don’t have to adopt healthy behaviors”. In addition, in some cross-sectional studies, many participants held incorrect beliefs or knowledge about the quality requirements, efficacy, and safety of functional foods [24,25]. These studies questioned participants beliefs about the effectiveness of functional foods themselves, such as “regular food supplement intake can prevent cancer”, and did not reveal whether people believe that functional foods will compensate for the negative effects of engaging in unhealthy behaviors or not engaging in healthy behaviors, and it is unclear how those false beliefs and knowledge are generated in people.

Beliefs that one behavior can compensate for the negative aspects of another behavior (e.g., “By taking functional foods, I can adopt unhealthy behaviors or I don’t have to adopt healthy behaviors”) are organized as compensatory health beliefs and have been discussed in other health-related behaviors. People can experience conflict between their goal to be healthy and their desire to behave unhealthily (e.g., to eat unhealthy food) or not behave healthily (e.g., not exercise). Previous studies showed that people will activate compensatory health beliefs (CHBs) to resolve this conflict [26,27]. CHBs are beliefs that “the negative effects of an unhealthy behavior can be compensated for, or “neutralized”, by engaging in another, healthy behavior” [26,28]. The authors present “I can eat this piece of cake now because I will exercise this evening” as an example [26]. When some people face conflict between their desires and goals, they have CHBs that the negative effects of unhealthy behaviors (e.g., eating fat-rich dishes) can be eliminated by the positive effects of compensatory behaviors they consider “healthy” (e.g., intake of functional foods) [26,28]. Previous research suggested that users of Meal Replacement Products (MRPs; i.e., products adjusted for nutrient composition that are consumed as meal replacements) have stronger CHBs about MRPs (e.g., “By consuming MRPs for weight regulation, one can compensate overeating at the previous meal”) than non-users [29]. However negative health outcomes will result if these compensatory behaviors are ineffective [26,27]. Therefore, when people consume functional foods rather than engaging in healthy behaviors, it could result in negative health outcomes.

In terms of fat-reduction functional foods, audiences may try to avoid the conflict between the desire to eat fat-rich dishes (e.g., steak, ramen) and the goal of managing their weight and health through the compensatory behavior of taking functional foods. This may mean they believe that they can achieve their goal while satisfying their desire. In the present study, we considered the belief that “By taking functional foods, I can adopt unhealthy behaviors or I don’t have to adopt healthy behaviors” as functional foods related to CHBs (FF-CHBs). It is reasonable to assume that functional foods do not compensate for the negative effects of unhealthy behaviors and the absence of healthy behaviors. Therefore, the activation of FF-CHBs (i.e., “By taking functional foods, I can adopt unhealthy behaviors or I don’t have to adopt healthy behaviors”) is expected to result in negative health outcomes.

### 1.3. Aim and Hypotheses of the Present Study

To our knowledge, no studies have focused on the effects of exposure to functional foods ads on audiences. This study aimed to assess the effects of exposure to “Exemption” ads for fat-reduction functional foods on audiences. We developed the following two hypotheses.

1. Exposure to “Exemption” video ads increases participants’ belief that “By taking functional foods, I can adopt unhealthy behaviors or I don’t have to adopt healthy behaviors” (FF-CHBs).
2. Exposure to “Exemption” video ads increases participants’ intention to take functional foods.

## 2. Materials and Methods

### 2.1. Participants and Design

**Figure 1:**
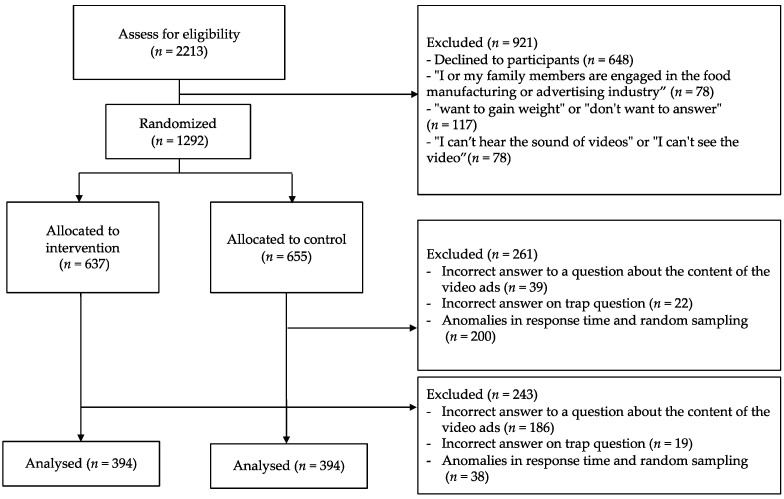
Participant flow.

We conducted a web-based randomized controlled study. Participants responded to a survey via their personal computers or smart phones on 29–30 September 2021. Participants were recruited from people registered with a survey company database in Japan. Emails were sent to registered users who responded to screening questions. The inclusion criteria were males and females aged 18–64 years. People who answered questions about their weight goals with “want to gain weight” or “don’t want to answer” were excluded (i.e., people who answered “Want to maintain weight” and “Want to lose weight” were included) to create a population with a common health goal. In addition, people who indicated that they or their family members were engaged in the food manufacturing or advertising industries were excluded because they could be familiar with video ads and functional foods, and the effects of the intervention on ad viewing could differ from the general population. Finally, those who answered “I can’t hear the sound of videos” or “I can’t see videos” were also excluded. People who were eligible and consented to participate were invited to complete a web-based survey. Participants were randomly assigned to a group that received an intervention video or a group that received a control video using an algorithm included in the survey program. After the random allocation, we screened participants to exclude lax participants. We asked participants about the content of the video to determine if they had viewed the video. We also used a trap question that required reading the question carefully. Participants who answered these questions incorrectly were excluded from the analysis. We also excluded those whose response time was abnormal (e.g., answered too quickly). From the screened participants who completed the survey, 394 participants each for the intervention and control groups were randomly selected by the algorithm. Figure 1 shows the participant flow.

This study was registered with the University Hospital Medical Information Network Clinical Trials Registry (UMIN-CTR) as a clinical trial (Unique Trial Number: UMIN000045393) on 6 September 2021. The methods used in the present study adhered to CONSORT guidelines [30]. The protocol was approved by the Ethical Review Committee of the Graduate School of Medicine, The University of Tokyo (number 2021179NI). All participants gave written informed consent in accordance with the Declaration of Helsinki.

### 2.2. Intervention and Control Videos

Participants in the intervention group viewed three videos with a total time of 50 s, and control group participants viewed two videos with a total time of 50 s. Based on our previous study [16], we extracted the three videos with the highest number of views per month from among the “Exemption” video ads (defined as “Taking the product frees the audience from refraining from binge eating”) for the intervention group. For example, one of the intervention ads showed fat-rich dishes, and the main character expressed her resistance to eating them. Then, the functional food was shown; the main character took the functional food and then enjoyed the fat-rich dish. We replaced the ad that had been removed from YouTube after our previous study with an ad with similar characters and themes. The company’s official YouTube channel had posted the three videos. Two videos promoted beverages and one promoted functional food in tablet form. These three functional food products had been permitted by the Japanese Consumer Affairs Agency (CAA) as Foods for Specified Health Uses (FOSHU) or self-certificated by businesses to CAA as Foods with Function Claims (FFC) [31,32]. All products had functional labeling that claimed they “reduced the absorption of fat ingested in the diet”. We acquired two videos for the control group about how to brew green tea from a major beverage manufacturer’s official YouTube channel.

### 2.3. Measures

The primary outcome was FF-CHBs, namely “By taking functional foods, I can adopt unhealthy behaviors or I don’t have to adopt healthy behaviors”. The first scale to assess CHBs was developed in 2004. It was tested for validity and reliability and measured general CHBs for a variety of lifestyle behaviors, including sleep and stress. However, rather than measuring general CHBs, it has been suggested that such scales should be situation specific [33]. Several situation-specific scales were developed, including CHBs for tobacco [34] and breastfeeding [35]. Therefore, in this study, we adopted and modified a scale based on a Meal Replacement Products scale to focus on FF-CHBs [29].

Participants responded to the nine questions on a scale from 1 to 6 (“extremely unlikely”, “unlikely”, “a little unlikely”, “a little likely”, “likely”, and “extremely likely”). The two reasons for adopting the 6-point Likert were to align with CHBs research on MRP [29] and participants’ easy response to choose “neither”. This measure comprised two factors. Factor 1 included five questions about beliefs that “By taking functional foods, I can adopt unhealthy behaviors”. Factor 2 included four questions about beliefs that “By taking functional foods, I don’t have to adopt healthy behaviors”. Appendix A (Table A1) shows each question mean, SD, and factor loading. Before answering the nine questions, we presented participants with the following statement. “Please read each statement carefully and tell us to what extent you think so or do not think so. Note that functional foods in this question refer to functional foods such as supplements and teas that are labeled as “reducing the absorption of fat and sugar”.

The secondary outcome was intention to take functional foods [36]. Using the format “I intend to perform goal-directed behavior ‘y’ when I encounter situation ‘z’”, we asked participants whether they intend to consume functional foods when an excess of fat-rich dishes occurred. Participants responded to two questions on the scale from 1 to 6: “I would take functional foods when I eat too much fat in my previous meal” and “I would take functional foods if I ate too much fat the day before”.

These questions were measured before and after participants were exposed to the intervention or control videos, following a research design related to health communication studies [37,38]. Mean scores (from 1 to 6) were used for the analysis. Higher scores indicated stronger FF-CHBs (i.e., belief that “By taking functional foods, I can adopt unhealthy behaviors or I don’t have to adopt healthy behaviors”) and greater intention to take functional foods. In addition, all participants were asked for sociodemographic information before exposure to the intervention or control videos. To clarify the characteristics of the target population, we also asked intervention participants about the number of times they had seen the three intervention ads.

### 2.4. Sample Size

To our knowledge, no previous intervention study has examined the effects of advertising exposure on CHBs. Based on a summary of social psychological research effect sizes over a century [39], we estimated an effect size (Cohen’s d) of 0.2 in the present study. We conducted a power analysis at an alpha error rate of 0.05 (two-tailed) and a beta error rate of 0.20. The power analysis indicated that 394 participants each were required in the intervention and control groups.

### 2.5. Statistical Analysis

Cronbach’s α values were used to determine the internal reliability of the measures. The factor structure of FF-CHBs scale was confirmed by conducting an exploratory factor analysis. Descriptive statistics were used to describe participants’ sociodemographic information, with percentages for categorical variables and mean (standard deviation) for continuous variables. Unpaired t-tests were conducted with the mean change in values before and after for each measure as the dependent variable and group assignment as the independent variable. Student’s t-tests were performed when the assumption of homogeneity of variance was satisfied. Welch’s t-tests were performed when the assumption of homogeneity of variance was not satisfied. A strong correlation between the baseline value and the outcome is expected [40], therefore, we conducted an analysis of covariance (ANCOVA) with the baseline values of outcome as the covariate. η^2^ was calculated to determine effect size and then converted to Cohen’s d [41]. A *p*-value of <0.05 was considered significant in all statistical tests. All statistical analyses were performed with R (version 4.0.3).

## 3. Results

### 3.1. Participants’ Characteristics

Table 1 shows participants’ characteristics. In total, 45% of participants were male. Participant age ranged from 18 to 64 years (mean = 40.0, SD = 11.2). The mean (SD) BMI was 22.5 (4.11), and 68% of the participants had a BMI of 18.5–24.9. About 70% of participants wanted to lose weight as their weight management goal. In addition, 54% of participants had an educational attainment beyond college graduation and most (95%) participants had not received advice from health professionals about their diet. Of the 394 participants in the intervention group, 117 (29.7%) had seen all three videos used in the intervention in their daily lives, and 107 (27.2%) had never seen any of these videos.

### 3.2. Comparison of Outcomes between Groups

The Cronbach’s α values for the internal consistency of the two outcomes were: 0.913 for FF-CHBs (“By taking functional foods, I can adopt unhealthy behaviors or I don’t have to adopt healthy behaviors”) and 0.936 for intention to take functional foods.

Table 2 shows the comparisons of outcomes between groups. The change in FF-CHBs in the intervention group was significantly greater than that in the control group (mean = 0.16 vs. −0.10, *p* < 0.001, Cohen’s d = 0.528). The change in intention to take functional foods was significantly greater in the intervention group than in the control group (mean = 0.21 vs. 0.06, *p* = 0.018, Cohen’s d = 0.169).

Analysis of covariance with the baseline FF-CHBs set as the covariate revealed the main effect of the group assignment (F (1, 785) = 56.849, *p* < 0.001, η^2^ = 0.026, Cohen’s d = 0.324). Analysis of covariance with the baseline intention to take functional foods set as the covariate revealed the main effect of the group assignment (F (1, 785) = 4.957, *p* = 0.026, η^2^ = 0.003, Cohen’s d = 0.109). The estimated means of FF-CHBs and intention to take functional foods after intervention with the covariate adjustment for baseline values are shown in Table 3.

## 4. Discussion

### 4.1. Discussion

Our results showed that exposure to “Exemption” functional foods video ads increased participants’ FF-CHBs (i.e., belief that “By taking functional foods, I can adopt unhealthy behaviors or I don’t have to adopt healthy behaviors”), and intention to take functional foods. Therefore, our results supported our hypothesis.

Regarding the increase in FF-CHBs, the change in the FF-CHBs in participants who were exposed to the “Exemption” ads between before and after the intervention was significantly greater than that in the control group. The observed effect size (Cohen’s d) was 0.528, which was considerably larger than values reported in previous social psychology studies [39,42]. One intervention video showed a fat-rich dish; the main character expressed resistance to eating the dish and consumed the functional foods. This ad claimed that the conflict between the desire to freely consume fat-rich dishes and the goal to resist consuming them can be resolved by functional foods. Previous literature on CHBs showed that people used three strategies to mitigate the conflict between a desire and a goal [26]. The first strategy was adapting the perception caused by behavior and re-evaluating outcome expectancies (i.e., adjusting the outcome expectation so that eating some fatty food was not negative for health, or changing the risk perception so that eating some fatty food was acceptable). The second was deciding to resist the desire. The third was activating CHBs. Based on the theory of CHBs, our results can be interpreted as indicating the content of the video ads, which presented functional foods as an easy solution for the conflict between desires and goals, promoted active FF-CHBs (i.e., “By taking functional foods, I can adopt unhealthy behaviors or I don’t have to adopt healthy behaviors”).

Previous studies indicated that weight-loss supplements intake can lead to unhealthy behavior [20,21,22,23]. One study found that participants in the weight-loss supplements condition consumed more nougat and preferred more sugar in tapioca than those in the control condition [21]. The results of the present study are consistent with the results of the previous study. We can also explain previous studies as a result of the activation of the FF-CHBs regarding the weight-loss supplements that the participants formulate daily, including ads [21].

Several situation-specific scales were developed, including CHBs for tobacco [34] and breastfeeding [35]. The present study also suggests that CHBs can be useful for psychological explanations related to functional foods, although the reliability and validity of the scale need to be tested in future studies.

The ratings for FF-CHBs in this study tended to be “unlikely” and “a little unlikely”, even after the intervention. This result was consistent with a previous study of users of MRPs [29]. However, we should not ignore this result because the changes in FF-CHBs in this study were generated from only one exposure to each of the three ads. Audiences are exposed to such ads many times daily. Furthermore, ads disseminated via TV and video distribution sites are viewed by a considerable number of people. In fact, two of the videos used in this study were viewed over 1.5 million times per month on YouTube alone. We found that even a single exposure to “Exemption” ads increased the FF-CHBs compared with no exposure. Although the changes in the measure were small, we must consider the effects of repeated exposure of many people to ads in TV and video streaming sites.

Regarding the increase of intention to take functional foods, the amount of change in intention to take functional foods among participants who were exposed to the “Exemption” ads was significantly greater than that in the control group. In this study, we were unable to measure the actual audience behavior, but measured the intention to take functional foods as a feasible alternative measure. If taking functional foods (i.e., compensatory behavior) does not compensate for the negative effects of unhealthy behaviors or not engaging in a healthy lifestyle, then negative health outcomes are expected [26,27]. Functional foods do not have the kind of effect that “Exemption” ads claim (“By taking functional foods, I can adopt unhealthy behaviors or I don’t have to adopt healthy behaviors”). Therefore, these “healthy” foods may lead to “unhealthy” outcomes because taking functional foods may not compensate for the negative effects of a fat-rich dishes. From the perspective of public health, it is necessary to improve the content of “Exemption” functional foods video ads.

### 4.2. Implications for Public Health Institutions and Government

In 2020, the CAA established a guideline stating that whether or not ads are misleading is judged from the impressions and perceptions from the ads as a whole, not from specific words or images alone [9]. The US Federal Trade Commission also issued the same caution regarding ads [43]. Although “Exemption” ads in this study did not clearly state that “By taking functional foods, I can adopt unhealthy behaviors or I don’t have to adopt healthy behaviors”, the ads as a whole provided a misleading impression to audiences. Some public interest foundations hold workshops for businesses on appropriate food labeling, including advertising for functional foods [44,45]. In these workshops, it would be helpful to specifically show what kinds of ads are likely to mislead audiences. The theory of CHBs will be useful to clarify the type of deceptive ads. “Exemption” ads create a conflict between unhealthy desires and health-related goals, and present functional foods as a solution to this conflict. In the case of functional food products that are designed and advertised as support for health goals, once the depiction of unhealthy desires is added, it will increase the conflict between desire and health goal, and then activate false FF-CHBs (i.e., “By taking functional foods, I can adopt unhealthy behaviors or I don’t have to adopt healthy behaviors”). Governments can regulate the content of inappropriate ads, such as “Exemption” ads, by prohibiting the depiction of unhealthy desires (e.g., images and audio to appeal the deliciousness of fat rich dishes). Structuring the content of ads based on CHBs theory may help to effectively regulate inappropriate ads for functional foods.

Functional foods and their advertising can also be seen as resources for public health. In some cases, functional foods and their ads may increase people’s interest in health. In our previous content analysis of functional foods video ads, we also identified “Lifestyle” ads (defined as “adding product intake to a healthy lifestyle”) [16]. “Lifestyle” ads, which depict physical activity of three metabolic equivalents or more in a positive light, accounted for seven of the 82 ads analyzed (8.5%) and had the highest average number of views per month. For example, one “Lifestyle” ad depicted smiling celebrity characters who were actively walking to achieve good health. The content of the “Lifestyle” ads was based on appropriate physical activity. “Lifestyle” ads do not contain the states of conflict between the desire to eat and the health goal and are unlikely to cause FF-CHBs that “By taking functional foods, I can adopt unhealthy behaviors or I don’t have to adopt healthy behaviors”. Although the purpose of advertising is to promote product sales, an increase in “Lifestyle” ads would be useful in terms of health promotion. To enable businesses to create such ads on their own initiative, the government and public health institutions should not only regulate the content of functional foods ads but also collect and share with businesses good practices, such as “Lifestyle” ads.

### 4.3. Implications for Businesses

Businesses that release “Exemption” ads need to actively participate in the above-mentioned workshops to improve their advertising. Furthermore, businesses should not focus on how to circumvent the law or guidelines, but rather on how they can integrate functional foods into the healthy lifestyle of their audiences. The MHLW advised the public that “A good way to use functional foods is to use them as a trigger for improving your diet and lifestyle” [17]. Businesses should take the initiative and find “a good way” to use their functional food products to improve lifestyle habits and reflect these aspects in their brand concepts and ads as the strength and originality of each functional food product.

### 4.4. Limitations

Several limitations of this study should be considered. First, the FF-CHBs scale was created based on a scale used in previous studies, but its reliability and validity need to be verified in further research. Second, because we conducted this study online, we were not able to monitor whether participants fully completed the task of viewing the videos and understanding the questions. To minimize this limitation, we included questions to exclude lax participants and those who had not viewed the ads and adopted a system that required clicking on the video link to proceed to the next question. Third, further studies should examine how the increase in FF-CHBs affect actual behavior. The participants in this study answered the questions just before and after watching the video, so the long-term effects remained unknown. Long term effects and effects of multiple exposures using a longitudinal design should be conducted in next research. Fourth, it is unclear to what extent the findings of this study can be generalized to populations other than participants in this study. However, this is the first study to assess the effects of exposure to functional foods video ads on the audience using the CHBs theoretical framework. This study offers important implications for the improvement of dietary environment and lifestyle behaviors through improving functional foods advertising in Japan.

## 5. Conclusions

This study assessed the effects of exposure to “Exemption” functional foods video ads, which are widely viewed in Japan. The main finding of this study is exposure to “Exemption” video ads increases participants’ FF-CHBs (i.e., “By taking functional foods, I can adopt unhealthy behaviors or I don’t have to adopt healthy behaviors”). Repeated daily exposure to “Exemption” ads via TV and YouTube may encourage audiences to adopt unhealthy behaviors and avoid healthy behaviors. The deceptive content of “Exemption” functional foods video ads should be improved to promote public health.

## Figures and Tables

**Table 1:** Participants’ characteristics.

	Intervention (*n* = 394) *n* (%)	Control (*n* = 394) *n* (%)	Overall (*n* = 788) *n* (%)
Gender, male	166 (42.1)	190 (48.2)	356 (45.2)
Age, years			
18–29	96 (24.4)	93 (23.6)	189 (24.0)
30–39	105 (26.6)	95 (24.1)	200 (25.4)
40–49	114 (28.9)	121 (30.7)	235 (29.8)
50–59	60 (15.2)	64 (16.2)	124 (15.7)
60–64	19 (4.8)	21 (5.3)	40 (5.1)
BMI, kg/m^2^			
<18.5	40 (10.2)	53 (13.5)	93 (11.8)
18.5–25.0	275 (69.8)	261 (66.2)	536 (68.0)
≥25.0	79 (20.1)	80 (20.3)	159 (20.2)
Weight management goal			
Want to gain weight	0 (0.0)	0 (0.0)	0 (0.0)
Want to maintain weight	112 (28.4)	131 (33.2)	243 (30.8)
Want to lose weight	282 (71.6)	263 (66.8)	545 (69.2)
Don’t want to answer	0 (0.0)	0 (0.0)	0 (0.0)
Highest education level			
Less than high school	7 (1.8)	7 (1.8)	14 (1.8)
High school graduate	85 (21.6)	96 (24.4)	181 (23.0)
Some college	84 (21.3)	77 (19.5)	161 (20.4)
College graduate	189 (48.0)	186 (47.2)	375 (47.6)
Graduate school	26 (6.6)	24 (6.1)	50 (6.3)
Household income			
Less than 2 million yen ^a^	41 (10.4)	48 (12.2)	89 (11.3)
2–6 million yen	202 (51.3)	176 (44.7)	378 (48.0)
More than 6 million yen	151 (38.3)	170 (43.1)	321 (40.7)
Participants with dietary treatments			
	15 (3.8)	20 (5.1)	35 (4.4)
Number of ads previously seen			
0	117 (29.7)		
1	82 (20.8)		
2	88 (22.3)		
3 (all)	107 (27.2)		

^a^ 1 US dollar is roughly equivalent to 100 yen. BMI, body mass index.

**Table 2:** Comparison of outcomes between the control and intervention groups.

	Intervention (*n* = 394)			Control (*n* = 394)			*p*-Value	Effect Size (d)
	Before	After	Change (After–Before)	Before	After	Change (After–Before)		
FF-CHBs	2.20 ^a^ (0.77) ^b^	2.36 (0.85)	0.16 (0.11–0.22) ^c^	2.22 (0.73)	2.12 (0.74)	−0.10 (−0.14–−0.06) ^c^	<0.001 ^d^	0.528
Intention to take functional foods	2.87 ^a^ (1.15) ^b^	3.08 (1.22)	0.21 (0.12–0.30) ^c^	2.94 (1.18)	3.00 (1.20)	0.06 (−0.02–0.14) ^c^	0.018 ^d^	0.169

^a^ Mean. ^b^ Standard deviation. ^c^ 95% confidence interval. ^d^ *p*-values for comparing amount of change among intervention groups using t-test. FF-CHBs, functional foods-related compensatory health beliefs.

**Table 3:** Estimated means of FF-CHBs and intention to take functional foods after intervention with the covariate adjustment for baseline.

	Intervention (*n* = 394)	Control (*n* = 394)	*p*-Value	Effect Size (η^2^)
FF-CHBs	2.37 ^a^ (2.32–2.42) ^b^	2.11 (2.06–2.16)	<0.001	0.026
Intention to take functional foods	3.11 ^a^ (3.03–3.19) ^b^	2.98 (2.89–3.06)	0.026	0.003

^a^ Estimated means after intervention with the covariate adjustment for baseline. ^b^ 95% confidence interval. FF-CHBs, functional foods-related compensatory health beliefs.

## Data Availability

The data presented in this study are available on request from the corresponding author.

## Appendix A

**Table A1:** Factor loading values of functional foods related to Compensatory Health Beliefs (FF-CHBs).

Item	Mean	SD	Factor Loading	
			F1	F2
**Factor 1 [By taking functional foods, I can adopt unhealthy behaviors].**				
When I take functional foods after a meal, I can eat more fat-rich foods.	2.34	0.97	**0.81**	0.36
By taking functional foods, I can eat more fat-rich foods in the next meal.	2.34	0.97	**0.80**	0.36
By taking functional foods, I can make up for the excess fat intake in my previous meal.	2.57	1.07	**0.76**	0.26
By eating functional foods, I can make up for the excessive fat intake of the previous day’s meal.	2.41	1.03	**0.74**	0.32
If I take functional foods, I can eat a little too much fat in my diet.	2.47	1.02	**0.65**	0.26
**Factor 2 [By taking functional foods, I don’t have to adopt healthy behaviors].**				
If I take functional foods, I don’t need to eat fruits and vegetables to control my weight.	1.88	0.90	0.24	**0.76**
If I take functional foods, I don’t have to work out to control my weight.	1.88	0.93	0.29	**0.76**
If I take functional foods, I don’t have to restrict my diet to control my weight.	2.09	0.96	0.33	**0.76**
Taking functional foods can compensate for lack of exercise.	1.88	0.92	0.27	**0.71**

Factor loadings above 0.40 are marked in bold.
